# Mining biomedical images towards valuable information retrieval in biomedical and life sciences

**DOI:** 10.1093/database/baw118

**Published:** 2016-08-18

**Authors:** Zeeshan Ahmed, Saman Zeeshan, Thomas Dandekar

**Affiliations:** 1 The Jackson Laboratory for Genomic Medicine, Farmington, CT, USA; 2 Department of Bioinformatics, Biocenter, University of Wuerzburg, Wuerzburg, Germany; 3 EMBL, Computational Biology and Structures Program, Heidelberg, Germany

## Abstract

Biomedical images are helpful sources for the scientists and practitioners in drawing significant hypotheses, exemplifying approaches and describing experimental results in published biomedical literature. In last decades, there has been an enormous increase in the amount of heterogeneous biomedical image production and publication, which results in a need for bioimaging platforms for feature extraction and analysis of text and content in biomedical images to take advantage in implementing effective information retrieval systems. In this review, we summarize technologies related to data mining of figures. We describe and compare the potential of different approaches in terms of their developmental aspects, used methodologies, produced results, achieved accuracies and limitations. Our comparative conclusions include current challenges for bioimaging software with selective image mining, embedded text extraction and processing of complex natural language queries.

## Introduction

There has been an enormous increase in heterogeneous biomedical literature and image production in the last decades (1). There are many publically available databases (Supplementary Material, Table S1), which are keeping small and large biomedical image datasets of various kinds and from different research and clinical projects e.g. *Electron Microscopy Data Bank (EMDB); Whitney Imaging Center; COllaborative Informatics and Neuroimaging Suite (COINS); LONI image data archive; The Cancer Imaging Archive (TCIA); Grand Challenges in Medical Image Analysis, Alzheimer’s Disease Neuroimaging Initiative (ADNI); Open Access Series of Imaging Studies (OASIS); Breast Cancer Digital Repository (BCDR); Digital Database for Screening Mammography (DDSM); The Mammographic Image Analysis Society (MIAS); Mammography Image Databases (MID); NLM HyperDoc Visible Human Project color, CAT and MRI image samples; The Histology Image Dataset (histologyDS); The Cancer Genome Atlas (TCGA); International Cancer Genome Consortium, Stanford Tissue Microarray Database (TMA); MITOS dataset, Cancer Image Database (caIMAGE); DPA’s Whole Slide Imaging Repository; Atlas of Bleast Histology; Histology Photo Album, Tissue Acquisition and Banking Services (TABS) of the NYU Experimental Pathology Core Facilities, Aperio Images, HAPS Histology Image Database; ITK Analysis of Large Histology Datasets; BDGP images from the FlyExpress database; The UCSB Bio-Segmentation Benchmark dataset; Pap Smear database; BIICBU Biological Image Repository; RNAi dataset; Chinese Hamster Ovary cells (CHO) dataset; Endogenus mouse sub-cellular organelles (END) database; 2D HeLa dataset (HeLa) database; Allen Brain Atlas; Cell Centered Database (CCDB); The Encyclopedia of DNA Elements (ENCODE); The Human Protein Atlas; DRIVE: Digital Retinal Images for Vessel Extraction; El Salvador Atlas of Gastrointestinal Video Endoscopy Images and Videos of his-res of studies taken from Gastrointestinal Video endoscopy; BiMed; Public Image Databases; Dartmouth Biomedical Libraries; The National Library of Medicine presents MedPix; New Database Provides Millions of Biomedical Images, DrumPID, STRING* etc.

It is one of the highly complex and unaccomplished tasks of today to implement a system, which can help scientists and physicians in collecting, curating, annotating and validating information distributed among by biological databases and published scientific literature. There is no standalone system available, which can standardize annotation protocols and facilitate successful execution of complicated natural language based queries, as most of the available systems support in random data browsing, search by example, sketch, text and navigation using customized image classes. However, there are some running projects, which are applying different algorithms, deep neural networks and machine learning techniques to analyse different kinds of biomedical images to help scientists and physicians in understanding and predicting the behaviour of complex biological systems, e.g. *TensorFlow* by Google (https://www.tensorflow.org/), *The Medical Imaging Interaction Toolkit* (MITK) (http://mitk.org/wiki/MITK), *iHOP* (http://www.ihop-net.org/UniPub/iHOP/), Microscopy *Image Browser* (http://mib.helsinki.fi/) etc. To curate and improve biomedical image databases, such tools are important as otherwise the information pertaining to the figures is not properly separated from text parts, explaining experimental conditions with biological conclusions.

**Figure 1.**
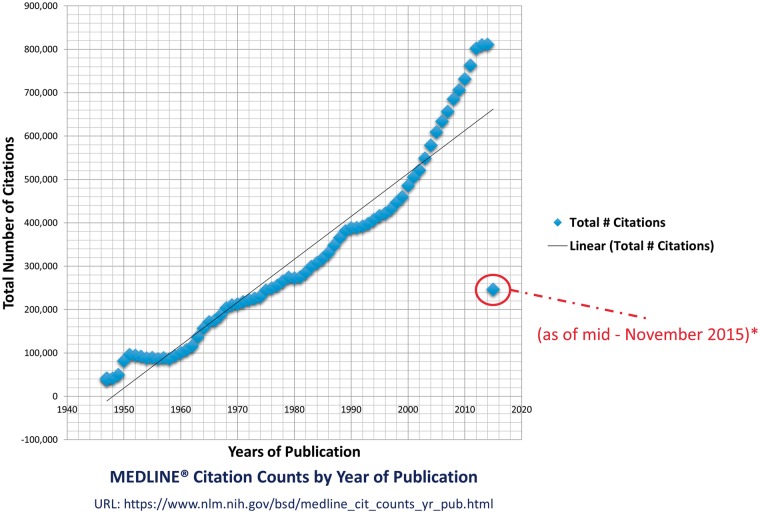
MEDLINE citation count. This figure shows the enormous increase in the citation count at MEDLINE over the last six decades. The year 2015s count is not complete but in progress. The graphed statistics are taken from the official website of the MEDLINE by the US national Library of Medicine (http://www.nlm.nih.gov/bsd/medline_cit_counts_yr_pub.html), attached in supplementary material.

So far, over 21 million high quality references have been indexed at MEDLINE database (maintained by the United States National Library of Medicine at the National Institutes of Health) from >5600 journals and in around 40 different languages (http://www.nlm.nih.gov/bsd/medline_cit_counts_yr_pub.html) (Figure 1). The cited literature at MEDLINE is connected to 38 biomedical databases (2) with literature about life sciences, preclinical sciences, medicine and health care systems. The user can access this literature using PubMed; an efficient information retrieval (IR) system with automatic term mapping and Boolean operators (3) to extract published literature at keywords and simple natural language processing (NLP) queries (e.g. titles, authors, abstracts, introduction etc.). The default outcome of a successful query at PubMed brings typically 20 relevant results per page; however, user can improve and customize search using advanced options. The applied search operators in PubMed are not robust and this can result into the extraction of un-expected articles, whereas, powerful search engines (e.g. Google Scholar) can lead to the correct articles with the same amount of information.

Orthodox IR approaches for biomedical literature are mostly text-based and with minimum focus on figures. This is why the performance accuracy of text-based approaches is far better than the approaches involving images, which produce numerous gaps in their results (4). In the last decade, many IR approaches and tools (5–29) have been reported (3), which apply different searching techniques to identify, classify, rank and cluster results. In this study, we have been mainly focused only on those IR and bioimaging approaches which have been proposed to search biomedical literature by analysing text, figures and text embedded in figures. We provide a comprehensive overview on different tools available for IR including direct explanations on different approaches, and usage of the software. In this sense, it is a database Meta tool that strongly enhances the capabilities of the interested reader in IR regarding biomedical data and publications.

## Materials and methods

To get a comprehensive overview on different mining approaches, we went through hundreds of manuscripts published by different communities (including NLP, semantic web, image processing and bioimaging). We found that several data mining solutions have been published over last 15 years but based on our focus; we have selected few of them to evaluate. The defined criteria for selecting literature included the consideration of most recent publications (preferably 2009–2015) in the field of bioimaging informatics. We queried publically available search engines (e.g. *Google, Yahoo* etc.), literature archiving and referencing databases (e.g. *PubMed, IEEE, ACM, Springer, Scopus, DOAJ, ARXIV and Google Scholar* etc.). We applied different keywords (e.g. *text mining, image mining, bioimaging, bioimaging informatics, biomedical text mining, exploring PubMed, retrieving textual information, finding diagrams* etc.) and short statements (e.g. *extracting text and images from biomedical literature, text extraction from biomedical images, biological image analysis, searching biomedical literature, optical character recognition (OCR) of biomedical images, biomedical image segmentation, NLP and biomedical image analysis, biomedical document retrieval, accessing figures from biomedical literature, domain specific image analysis, text and image mining, figure search from MEDLINE, parsing biomedical images, mining biomedical images and text, biomedical language processing, web-based image retrieval, pathway diagram analysis, protein–protein interaction diagram analysis, ontology and image retrieval* etc.) to find most relevant literature. While examining the novelty of extracted literature; we were focused on the potential, methodology and accuracy of presented approach as well as the authors (first and last) other related publications (if exists).

**Figure 2.**
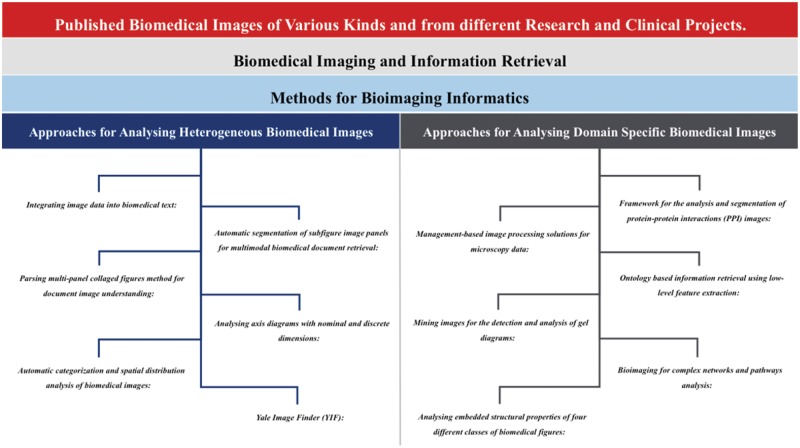
Concept of information extraction from published scientific and biomedical literature. This figure gives the overview of different processes involved in the information extraction from scientific and biomedical literature including data retrieval (getting text and figures from biomedical archives using NLP queries), information extraction (text mining and image processing) and presenting integrated data.

During this study, we went through different scientific NLP and IR approaches, which have been proposed for text and biomedical image mining e.g. *ImageJ* (30), *CellProfiler* (31), *CellPloc* (32), *Vaa3D* (33), *Icy* (34), *Konstanz Information Miner* (*KNIME*) (35), *Fiji* (36,37), *Framework for the analysis and segmentation of protein-protein interactions (PPI) images* (38), *Automatic segmentation of subfigure image panels for multimodal biomedical document retrieval* (39), *Ontology based information retrieval from medical Images using Low Level feature extraction method* (40,41), *Parsing multi-panel collaged figures method for document image understanding* (42), *mining images for the detection and analysis of gel diagrams* (43), *bioimaging for complex networks and pathways analysis* (44), *automatic categorization and spatial distribution analysis of biomedical images* (45,46), *analysing the embedded structural properties of biomedical figures* (47), *Yale Image Finder* (*YIF*) (48), *integrating image data into biomedical text* (49) etc. We also found some commercial applications (e.g. *Velocity, Amira*, *Cellomics* etc.), which are mainly programmed for the comprehensive coverage of image processing tasks (37) and not fully able to address new biological questions (31). After comprehensive evaluation of different approaches, we divided our conclusions into two categories: (1) approaches that have been proposed for all kinds of biomedical image analysis in IR and (2) methods for domain specific bioimaging in IR (Figure 2).

## Biomedical imaging and information retrieval

In biomedicine, images have been used for clinical decision support (CDS) and articulating scientific research findings (39). Numerous amount of heterogeneous figures have been published in scientific and biomedical literature, which includes the results obtained from different biological and medicinal experiments (e.g. *PCR-ELISA data, microarray analysis, gel electrophoresis, mass spectrometry data, DNA/RNA sequencing, diagnostic imaging CT/MRI and ultrasound scans*), medicinal imaging (e.g. *EEG, MEG, ECG, PET*) and other anatomical or pathological images. Biomedical images are published in biomedical literature following different classifications: flow charts, experimental images (e.g. *DNA, RNA and protein gel and microscopy images* etc.), models (e.g. *biological processes, experimental models, protein sequences, or higher protein structures* etc.), graphs (e.g. *line, spot and bar charts* etc.), image-of-thing (e-g. *cell, cell components, tissues, organs, species* etc.), mix tables and geometrical shapes (50). Graph and model images contain homogenous (non-texture) regions, whereas, experimental, image-of-thing, flow charts and geometrical shapes enclose texture. Most of the medical images are intensity only images, which bring lesser amount of information than colour images, however, colour images which are based on photography can bring more significant implications e.g. colour distribution can be helpful in detecting tumours (e.g. malignant tumour is severe reddish in comparison to the surrounding tissues whereas the benign tumour is not as intense), cancer (e.g. analysing skin colours in dermatology to interpret the characteristics of skin cancer) and anatomical organs. The precise analysis of these images can lead to the better understanding of different clinical and scientific problems.

Majority of the bioimaging approaches are domain specific, where different academic and commercial research organizations have been heavily contributed in implementing analytical systems for different kinds of experimental, medical and pharmaceutical images (51). Many approaches have been introduced to extract and analyse image features e.g. image annotation with maximum entropy to learn feature weights (52), colour-constant-colour-indexing (53), multi resolution histograms to distinguish between images with respect to the robustness and noise (54), medical image segmentation using Geodesic Active Contour (55), analysing image texture with visual information (56), medical image annotation (57) etc. The ultimate goal is to process images to identify their commonalities and variabilities for observatory scientific and clinical decisions, e.g. clinical assisting tool for analysing 3D brain images (58) and optical tomography image processing (59) etc.

Hundreds of bioinformatics tools have been developed and published, which can be useful in analysing heterogeneous lipidome to the genome data. In contrast, the work done in IR and document triage is far less. There are only few approaches (38,39,49,60–62), which can be useful in the classification, extraction and analysis of published data by processing NLP queries and image based text. It is one of the highly complex tasks and scientific challenges of current time to implement such system, which can efficiently extract embedded information (relevant to the clinical situations and experimental states) from published biomedical images (63). Most of the available NLP search engines focus only on the extraction and indexing of text from image captions (50). To enhance the IR mechanism, recently, it has been possible to partially extract the text from the biomedical images using different techniques (e.g. *classical image segmentation, automatically annotating images, image analysis for content-based image retrieval, supervised machine learning, automatic segmentation of subfigure image panels for multimodal biomedical document retrieval, ontology based information retrieval, mining gel images for analysis, analysing axis diagrams with nominal and discrete dimensions, mining of pathway diagrams, feature-based automatic categorization, structural feature-based analysis of image, histogram-based image processing, OCR* etc.) and search by combining with IMRAD (Introduction, Method, Results, Analysis and Discussion). Using partially extracted text from figures together with well-described legends, it is somewhat possible to understand the semantic and search with NLP but it is still a struggling question to perform NLP search based on the shapes of images.

During our study, we found that the figures are not well structured and archived in biomedical database as standalone entities and most of the times are only published inside the (PDF documents) articles (38). Moreover, non-scientific and irrelevant images (e.g. *journal logos, titles* etc.) are also embedded inside the published documents, which also decreases the efficiency in automatic images based content extraction and classification. The absence of structured and standardized ways of publishing figures in biomedical literature is a major reason for delays in implementing reliable systems for bioimaging informatics (64). It’s true that even with the availability of many new, modern and innovative approaches, it has not been completely possible to separate multi-panel figures, process complex pathways, extract biological components (e.g. *molecules, isoforms, genes, proteins* etc.) and predict their relationships. However, on-going IR research has somewhat contributed in the development of some new methodologies for biomedical image’s fractional text and feature extraction together with the combination of IMRAD.

## Methods for bioimaging informatics

**Figure 3.**
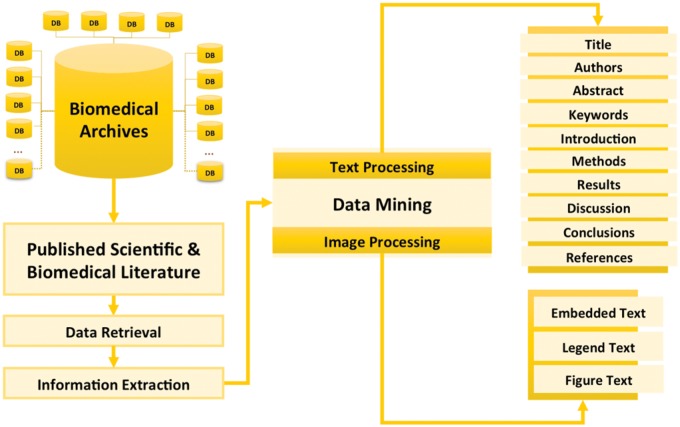
Concept of information extraction from published scientific and biomedical literature. This figure gives the overview of different processes involved in the information extraction from scientific and biomedical literature including data retrieval (getting text and figures from biomedical archives using NLP queries), information extraction (text mining and image processing) and presenting integrated data.

Theoretically, the process of hybrid computational information extraction from published scientific and biomedical literature is based on both text and image mining (Figure 3). Using NLP queries in some of available web and desktop applications, now, it has been possible to extract the most relevant literature of the reader’s choice from available libraries/databases.

Bioimaging informatics (65) is emerging in life sciences and medicine. Different methods have been proposed for the processing of gel images (66–68), analysing fluorescence microscopy images (69), extracting pathways (44), detecting axis (50), analysing cells structures to quantitatively measure phenotypes (31), quantitatively analysing 3D, 4D and 5D microscopic data (33) etc. Moreover, different feature extraction, segmentation and OCR based approaches have been proposed to identify specific text-based regions in documents (70).

Bioimaging informatics is somewhat similar to the classical literature processing, which includes four main methods: (1) document categorization, (2) named entity tagging, (3) fact and information extraction and (4) collection wide analysis (71). Whereas, the traditional IR process is based on only text mining to identify positive and negative document classification. Positive is the most relevant and negative is the most irrelevant document for annotation (49). Most of the times different classifiers have been trained and used to select and predict documents.

Published biomedical figures may include multiple panels (combination of several other figures). In such a situation, image segmentation is recommended to divide multi-panel digital images into multiple segments to trace objects and margins (72). Image segmentation is widely applied in different fields of life and sciences, e.g. content-based image retrieval (73), machine vision (74), object recognition and movement detection etc. Most bioimaging informatics approaches are also applying image segmentation with the help of different machine learning algorithms (36–39,42–46,48,49). Moreover, different methods (75–86) have been proposed to implement image segmentation (Table 1) and image retrieval system (87–91).

**Table 1.** Methods implementing image segmentation in IR

Method	Description	Limitations
Thresholding or Binarization (75,76)	This is a method based on the image segmentation, which create binary of gray scale images to perform image analysis. Various methods (e.g. point dependent techniques, region dependent techniques, local thresholding, multithresholding, Histogram Thresholding (77), Picture Thresholding (78), minimum spatial entropy threshold (79), Fuzzy entropy thresholding (80) etc.) have been proposed for thresholding.	Incorrectly set threshold can lead to under or over segmentation of objects (75).
Clustering	To understand large-scale complex data (text and images etc.), this method is widely applied in different fields (e.g. information retrieval, bioimaging, medicine etc.) for pattern recognition, speech analysis and information retrieval (81). To perform image features and text analysis, clustering divides content in possible numbers of meaningful group objects by breaking it into subcategories and draw relationships between them (82). There have been many different methods [e.g. Image segmentation by Clustering (83), Dual clustering (84) etc.], techniques (K-means clustering, Hierarchical clustering, Partitional clustering, Exclusive Overlapping clustering, Fuzzy clustering, Fuzzy C-means (FCM) clustering, Complete clustering, Partial clustering, Agglomerative Hierarchical Clustering, etc.) and types (Well-Separated, Prototype-Based, Graph-Based, Density-Based, Shared-Property etc.) for clustering.	It is difficult to predict fixed number of clusters while grouping objects and it consumes extensive computational time.
High Dimensional Indexing (HDI) (85)	There have been many HDI techniques proposed for large scaled content-based image retrieval, which have been categorized in Dimension Reduction [embedded dimension, Karhunen–Loeve transform (KLT), low-rank singular value decomposition (SVD) etc.], and Multi-dimensional indexing (Bucketing algorithm, priority k-d tree, quad-tree, K-D-B tree, hB-tree, R tree etc.) techniques (86).	Blind dimension reduction might not bring optimistic results during embedded dimension reduction.

One of the recent challenges is to combine figure captions and the embedded text inside figures with traditional IR process, and try to improve the hybrid IR mechanism. This can only be possible with the use of excellent OCRs and with the implementation of efficient image processing algorithms at figures presenting medicinal and experimental findings.

Feature analysis is another well-applied technique for extracting, measuring and analysing the features of images composed of variable objects or groups of objects (92). These variable objects can be different shapes representing drawings, organs, characters etc. In the case of characters, different OCRs (93) have been proposed and implemented which identify typewritten or printed text in the images. This is one of the widely used approaches in machine translation, text-to-speech, key data and text mining (94). The deployed process involves five main steps: (1) convert colour image to gray scale with different shades, (2) perform filtering, (3) extract features by analysing initial darker pixels, (4) recognize patterns by matching generated binary formats and (5) produce output based on the implemented system’s understanding.

Different machine learning techniques, Fourier descriptors (95) and neural network-based algorithms (96) have been proposed and implemented for the feature-based image processing and well applied in various fields of life, science and medicine. During our study we also found and discussed some domain specific and open bioimaging approaches (38,40,41,43,44,47,50) implementing feature extraction and OCR.

## Approaches for analysing heterogeneous biomedical images

The process of biomedical image analysis in IR is divided into three classes: (1) feature-based image analysis, (2) image segmentation and (3) text recognition using OCR. Whereas, biomedical image mining and text extraction approaches have been categorized into two groups: domain specific and open field. The open field approaches are those which focus on all kinds of biomedical images, whereas, domain specific approaches are targeting only precise images from different scientific and medicinal fields e.g. protein–protein interactions, clinical health care etc.

We collected and found a variety of different open field bioimaging approaches (e.g. automatic segmentation of subfigure image panels for multimodal biomedical document retrieval, Parsing multi-panel collaged figures method for document image understanding, analysing axis diagrams with nominal and discrete dimensions, automatic categorization and spatial distribution analysis of biomedical images and *YIF*) contributing to IR by automatic image segmentation, parsing the collaged figures with multiple panels, analysing axis, categorization and spatial distribution, implementation of histogram-based image processing etc.

### Integrating image data into biomedical text

This is one of the pioneer works for extracting textual information from images and combining output to the traditional IR mechanisms. Thus, Duygulu *et al.* (97) performed image feature-based analysis and biomedical document categorization based on the implementation of naïve Bayes classifier adopting content-based image retrieval concepts. This approach is divided into five steps: (1) figure extraction, (2) figure segmentation (98), (3) subfigure classification (classified into graphical, experimental and other classes, using support vector machine classifiers based on vectors with 46 features), (4) subfigure clustering into finer groups [using k-means algorithm, as implemented in Waikato Environment for Knowledge Analysis (99)] and (5) document representation as an image-based feature vector (49).

The approach was validated using a dataset of published articles (only from the Journals of Biological Chemistry, Journal of Cell Biology and Proceedings of the National Academy of Science) between 2002 and 2004. About 5837 articles were used to train classifier and 6043 articles were tested. Classifier was trained using 26 positive and 230 negative documents from JCB’02, which resulted in 1881 figures and 10 920 subfigures. Later, it was tested at 34 positive and 325 negative documents from JCB’03, which resulted in 2549 figure and 15 549 subfigures (97). Authors used evaluation metrics (100) to classify results, and computed precision, recall and F-score for the image-features system (0.279, 0.353 and 0.312), simple text classifier (0.647, 0.323 and 0.431), integrated (0.315, 0.5 and 0.386) and average of 59 runs (0.138, 0.519 and 0.195).

### Automatic segmentation of subfigure image panels for multimodal biomedical document retrieval

Particle swarm optimization (PSO) implements clustering algorithm for automatic segmentation of multi-panel images for multimodal biomedical document retrieval. PSO is categorized image processing in three phases: (1) Regular, (2) Illustration and (3) Mixed images (39). Regular applies basic concepts of image processing (converts RGB image to Grey, then calculates variance of vertical and horizontal lines across the image, then calculates the boundary edges with Grey horizontal and vertical dynamic range and in the end apply logical OR) to segment multi-panel images with a two-phase algorithm (by finding inter-panel boundary lines and input to train and test the neural network). Illustration applies three state algorithm [Sobel edge detector (98)] and forms bounding boxes to select five features (width, height, major axis length, minor axis length and axis ratio) for computing the fitness function of size and 14 features [solidity, extent and weighted density distribution features (100)] for shape. Mixed phase applies particle swarm optimization clustering (39). The reported limitations of PSO are that, it can only be applied to unimodal and individual images, which have to be manually segmented into individual panels.

The authors of PSO has claimed achieved accuracy rate of 94.9% for non-illustrative images and 92.1% for illustrative images (39). They validated approach at 1237 annotated medical images (756 non-illustrative and 481 illustrative) from five Biomed Central journals (Cancer, Advanced Fire Protection Systems (AFPS), Urology, Surgery and Cardiovascular Ultrasound). Moreover, they used structured vocabularies from the National Library of Medicine (NLM) and Unified Medical Language System (UMLS) to identify the biomedical concepts.

### Parsing multi-panel collaged figures method for document image understanding

This approach extract and classify figures from the biomedical literature by parsing the collaged multiple panel figures (fluorescence images, statistical plots and analysis procedure diagrams), splitting panels and creating single images (42). The overall methodology has been divided into two categories: (1) classification of figures (a process to identify if there is one or multiple figures in the text file), and (2) segmentation of figures (an iterative process of separating multiple panel figures to conclude with separate images in the end). While segmenting figures, authors have categorized them into photograph (illustrative figures) and non-photograph (all others) categories.

Using MATLAB, multivariate Gaussian functions and Hough transformation have been implemented to model normalized histograms (without profiling) and segment figures (42). Considering the unpredictable nature of panel margining in the figures from biomedical literature, authors did not apply the traditional, universal colour-based threshold method. Authors have implemented a two components based approach: (1) ‘half panel extraction’ (23) to it evaluate and separate long marginal figures (with homogenous horizontal and vertical arrays of pixels), and (2) ‘enclosed panel extraction’ to detect enlarged short margins to extract the enclosed panel using a Hough transformation (identifying the horizontal and vertical edges) and two heuristics (straight lines within the width and height of the panel at 0° and 90°).

To validate and test the accuracy of the implemented approach, authors have performed and reported two different experiments. First dataset was created which was based on 25 papers with 182 figures, and second dataset was based on 24 papers with 178 figures. Figures were automatically extracted (classified and segmented) with achieved average accuracy rate of 90% (dataset 1 = 91.8%, dataset 2 = 88.2%), with best empirical gray scale threshold (235). The reported error rate for all cases was <20% and for photographs it was <3% (42).

### Analysing axis diagrams with nominal and discrete dimensions

This approach focuses on the axis diagrams (50), representing nominal and discrete dimension of the underlying data. These kinds of diagrams are based on simple patterns and abundance in biomedical literature, known as the bar and line charts. Authors have performed image and caption data classification using WEKA (101) with a stemming algorithm (102), sequential minimal optimization (103), SVM (using the polynomial kernel) or segment-based algorithms. The overall approach has been divided into several steps: (1) retrieving figures as a bitmaps, (2) processing images (104), (3) segmenting with respect to size and shaped, (4) seeking possible labels, (5) eliminating cantered segments with less labels and (6) applying OCR to each extracted segment.

The authors have validated their approach with a small dataset of 100 randomly selected articles from PMC, containing 404 figures in total. They reported 89% achieved accuracy and claimed for better performance than Rodriguez-Esteban and Iossifov (105) (with 63–84%) and (106) (with 78.7%).

### Automatic categorization and spatial distribution analysis of biomedical images

This approach applies categorization and spatial distribution analysis of text to different kinds of biomedical images including flow charts, experimental, graph and mixed images. Authors have proposed and applied a new algorithm (46) to segment images by transforming images into binary, detecting all text regions using iterative projection histograms-based algorithm (104), extracting image features to categorize images with the help of SVM (45,46) in MATLAB using LIBSVM (107) library.

Authors have validated their approach using extracted images from PMC. In Ref. (45), authors tested 990 annotated images and estimated F-scores for each type (flow chart 0.9116, experiment 0.8211, graph 0.8398, mixed 0.7029, others 0.6514, conventional image features 0.489 and novel features 0.725). Following the same pattern, in Ref. (46), authors reported different results (*F*-scores: flow chart 0.9544, experiment 0.8770, graph 0.8857, mixed 0.7627, others 0.7778, conventional image features 0.489 and novel features 0.8581). The archived and reported results conclude with high accuracy [78.536% in (45) and 85.152 in (46)] in flowchart, experiment and graph images.

### Yale image finder

Yale image finder (*YIF*) is one of the pioneer approaches, publically available as a stable web-based search engine for retrieving the biomedical information by extracting the text from published biomedical literature at PMC (48). *YIF* authors have implemented histogram-based image processing (70) to perform customized layout analysis of images and extract text from images. They have applied crosschecking procedure at high-precision mode and skipping procedure at high recall mode to reduce the false positive results and indexed images using Apache’s Lucene.

According to the last reported figures (48), YIF has indexed over 140 000 images from >34 000 open access biomedical papers. *YIF* authors have validated their approach using 161 randomly selected biomedical images from the published literature at PMC. At high-recall mode they observed 64.79% image text content at 27.85% precision and 38.45% of the image text content at 87.68% precision at high-precision mode (48).

## Approaches for analysing domain specific biomedical images

Domain specific biomedical image analysis in IR is a lot more complex way of analysing biomedical images because it not only aims for extracting text from the images using OCRs but also analyses the structure of images to extract the semantic. During this study, we found very limited number of approaches (e.g. *Management-based image processing solutions for microscopy data, Framework for the analysis and segmentation of protein-protein interactions (PPI) images, Ontology based information retrieval using Low Level feature extraction, Mining images for the detection and analysis of gel diagrams, Bioimaging for complex networks and pathways analysis, Analysing embedded structural properties of four different classes of biomedical figures*) which analyses characteristics of domain specific biomedical images, performs feature-based analysis, segment multi-panel images, extracts text and implements Ontology.

### Management-based image processing solutions for microscopy data

*Fiji* is a cross-platform, standalone and open source framework. It has been developed in Java programming language using 3D libraries for surface extraction, enhanced volume rendering and interactive volume editing of three and four-dimensional biological and biomedical images. *Fiji* is an enhanced and compatible plugin for *ImageJ* (36), which have been developed as an excellent combination of modern software engineering principles, libraries, scripting languages and image-processing algorithms (37). It offers management-based image processing solutions towards registration, segmentation, measurement, annotation and visualization of large electron microscopy data. It is a well-documented application, which supports a broad range of scripting languages including *Clojure, Jython, Javascript, JRuby, Beanshell* etc.

Fiji allows direct display of image volumes, applies marching cubes algorithm to find a surface with desired threshold value and orthoslices represent three perpendicular and adjustable planes (36). It offers various image editing features, e.g. region of interest (ROI) tools (rectangles, ellipses and polylines), changing voxel values, annotation in 3D space, landmark-based 3D rigid registration of image volumes, automatic segmentation of image stacks and 4D visualization etc.

*Fiji* is not an application that provides any direct contribution to IR but offers flexible, open source features in using it for multiple purposes, e.g. as it is an open source application, one can use this platform for the analysis of large electron microscopic data images and can improve its features to use observed information in IR system.

### Framework for the analysis and segmentation of protein–protein interactions (PPI) images

It is one of the finest works in accessing text and image based information from the biomedical literature, with focus on protein–protein interactions (PPIs). This system is developed for the extraction of unimodal panels from full text articles (PDF files) by automatically extracting figure-captions to identify the numbering style and number of panels in the figures. Authors have divided their approach into eight modular components: (1) PDF operator parser (extracts layouts and captions), (2) figure filtering (eliminates irrelevant images, e.g. publisher’s logos etc.), (3) caption filtering (eradicates inaccurately harvested captions), (4) figure-caption matcher (links correctly identified figures to their respective captions), (5) caption segmentation (dividing captions in sub-captions to get the information about the specific panels of the figure), (6) image pre-processing (pixel-level processing to separate image objects from the background), (7) in-image text processing (lexical analysis on the extracted text from image) and (8) panel segmentation (using the output of caption segmentation, figure-caption matcher, image pre-processing and in-image text processing, to segment the figures into panels) (38). Together with their own innovative modular approach, authors have endorsed some existing approaches as well, e.g. they have used Xpdf tool (http://www.foolabs.com/xpdf) to extract full text from the PDF files, used OCR (ABBYY) similar approach to Xu *et al.* (48) to extract embedded text inside figures, used similar approach to (108) in caption segmentation and used the method (109) to compute the intensity value to distinct foreground region.

Authors have implemented their approach using a dataset of 2256 randomly selected full-text articles with 13 435 figure-caption pairs divided in 41 341 panels from the annotated corpus provided by the *MINT* database (110). Authors have compared produced results against an annotated gold standard by biologists (38) and claimed achieved accuracy rate of 96.64%. Along with the archived high accuracy rate, the authors also reported incorrect estimation of panels at 61.36%, incorrect estimation of connected components (CC) at 18.20% and region associated to incorrect panel at 20.44%.

### Ontology based information retrieval using low-level feature extraction

This approach has been proposed to reduce the semantic gaps between the textual descriptors and low-level features of the images (41) by combining ontology with low-level feature extraction method to retrieve information from the clinical health care domain. To analyse specific a ROI (111), authors have applied both context-based image retrieval (CBIR) (112) and region-based image retrieval (RBIR) (113) techniques. They have also applied different colour descriptors to identify the colour distribution and perform pixel-based analysis.

Authors have implemented their approach as a new IR system in an electronic health records (EHR) system to extract text and image-based information at NLP queries. Created the ontology for EHR has been divided into textual (details of the patient, doctor and health care facilities used by the patient) and feature (colour distribution, colour histogram and the region of interest) descriptions. The implemented system provides four searching methods: (1) retrieving EHR with respect to organs, (2) low-level features, (3) traditional searching and (4) by adding EHR to the database. The workflow of the implemented system starts with the textual instructions from the user or textual values extracted from the images, then creates and runs SPARQL query and in the end retrieves a list of URIs (individual records) (40,41). Authors developed graphical user interface in Java, created ontology in RDF and OWL using Protégé and most importantly used *ImageJ* to extract features from colour images. These authors successfully tested their application on a health care database by creating ontology but did not report the achieved accuracy rate. Some of the limitations of this approach are: this application works only on images with homogenous colour distribution, textual ontology is not well-structured as it can make misperceptions between the similar names of patient and doctor and even with hospital name.

### Mining images for the detection and analysis of gel diagrams

This approach is about processing gel images and extracting experimental results based on the protein expressions and protein–protein interactions (43). The overall designed and implemented system is divided into seven steps: (1) figure extraction, (2) segmentation, (3) text recognition, (4) gel detection, (5) gel panel detection, (6) named entity recognition and (7) relation extraction (43). Furthermore, image classification scheme is divided into five additional classes: (1) experimental or microscopy, (2) graph, (3) diagram, (4) clinical and (5) picture.

Authors have applied their approach to a large number of datasets, consisting in total of 410 950 articles; out of these, they were able to process 386 428 articles, accessed 1 110 643 figures, processed 884 152 figures, detected 85 942 gel panels, identified 0.097 gel panels per figure, spotted 309 340 gel labels (per panel 3.599), detected 1 854 609 gene tokens (75 610 gel labels, ratio 0.033 and ratio in gel labels 0.068) (43).

Unlike most of the existing and some of the mentioned approaches in this study, images are not mined from the published biomedical literature (PDF files) but extracted only from the structured XML files, available at the PMC database with additional image files. An iterative text detection algorithm (104,114) has been applied to detect the layouts (elements and edges) and text has been extracted using Microsoft document imaging OCR. Authors have implemented machine learning algorithms with 39 defined numerical features, 16 gray scale histogram features and 13 texture features RGB colour features (115). They have also used positioning coordinates, relative and absolute width and height values and some recognized characters (43). Authors used the *WEKA* toolkit (116) and opted for random forest classifiers at different thresholds, and tested the dataset of 500 randomly selected and manually annotated (gel segment) figures. They have applied different machine learning (ML) algorithms including naive Bayes, Bayesian networks (117), PART decision lists, convolutional networks but well concluded with random forests (43). Targeting high-precision gel panel detection, the authors tried to find the gel regions forming the central part of the gel panel and then extracted text labels around the panel.

The authors prepared a dataset of 3000 manually annotated figures, extracted from the published biomedical literature (accessed from PMC), structured in XML format. The achieved F-score in balanced conditions is 75%. The found limitations include the detection of the nearest neighbour in identifying the text label up to the range of 150 pixels maximum and unable to present solid results from the relation extraction.

### Bioimaging for complex networks and pathways analysis

This approach is about implementing a new bioimaging system for complex biological network diagrams’ analysis, data extraction, search, comparison and management (44). The proposed system’s methodology has been divided into seven steps: (1) pre-processing, (2) objects recognition, (3) relationships recognition, (4) filtering non-pathway images, (5) text tokenization, (6) ontological analysis and (7) filtering non-relational objects.

Authors have implemented a system in two integrated phases: (1) performed preliminary image processing (118) and (2) text recognition. Doing image processing, they first scanned biological and ontological terms using *IntegromeDB*, then with the application of mathematical morphology and binary analysis routines extracted objects and their relationships using ImageJ (30), then transformed the colour image to gray scale (32-bit RGB) using Daum–Huang particle filter (119), applied mathematical morphology (120) to eliminate small areas and in the end extracted all possible objects with particle analysis. Later, authors applied CuneiForm OCR for batch text recognitions, AutoIt-v3 for automatic batch operations and used *Lucene* for indexing and ranking of the text. The authors tested their approach at over 150 journals, with 50 000 articles and processed around 25 000 pathway figures from the biomedical literature available in PMC and the web. With the consent and involvement of biological experts, authors concluded their system with around 87% of accuracy.

### Analysing embedded structural properties of four different classes of biomedical figures

This approach is about analysing embedded structural properties of four different classes (charts, plots, geometrical shapes) of figures and biomedical images from published biomedical literature, using retrieval-based pattern approach (47). Authors have analysed differences in structural information based on binary representation and utilized the intensity edge statistics of annotated, compressed and enhanced figures. They used Canny edge detector for strong and weak edge detection; Fourier Edge Orientation Autocor-relogram (FEOAC) for noisy edges; edge orientation histogram (EOH) for analysing the distribution of edge pixels; histogram of radon transform (HRT) for the retrieving binary trademark and structural images; and R-signature (R-SIG) to distinguish binary shapes matching to the real life objects.

The authors have validated this approach using different threshold values on 180 published biomedical figures (49 diagram, 40 plot, 42 biomedical images and 49 binary shapes) from 73 biomedical articles. They observed low performance rate at both moderate (30%) and high (50) threshold for all five methods (FEOAC, EOAC, EOH, HRT and R-SIG) (47).

## Discussion

As we know that published text is not the only way of expressing information but figures and videos are also playing a dynamic role in biomedical and scientific content presentation. This is one of the reasons; why it is important to consider and combine embedded text in figures and videos, especially when creating IR systems for searching biomedical literature. It is one of the extremely complex tasks to implement a system, which can analyse all kinds of scientific images and report semantic in textual format to improve the IR mechanism. There are many domain specific bioimaging-based methods, which can produce efficient results with high accuracy rates but unfortunately there are only a few methods, which can extract text and analyse important features from all kinds of biomedical images. Going a step further, we found that the archived accuracies and performance levels of the open field IR approaches are higher than the domain specific IR approaches.

During this study, we found, analysed and reported on various bioimaging informatics approaches, which are partially helping the biomedicine communities in searching published literature (comparative overview of key strategies in Table 2). It is not directly possible to compare different open source bioimaging informatics approaches, as the observed results can be heavily lopsided by how the implemented application has been adjusted and used. We determined that all found approaches were well-proposed and published but with different positive aspects e.g. integrating image data into biomedical text can be well-applied in the extraction of textual information from images; PSO and Parsing multi-panel collaged figures method for document image understanding can be efficient in automatically segmenting subfigure images; Analysing axis diagrams with nominal and discrete dimensions can be helpful in doing analysis of the images with nominal and discrete dimension of the underlying data; and *YIF* can be used for text extraction and customized layout analysis of biomedical images. Moreover, we found *Fiji* is an interesting approach based on modern software engineering principles for the analysis of management-based images; framework for biomedical figure segmentation and mining images can be useful for the detection and analysis of complex protein–protein interactions; low-level feature extraction with ontology can be useful for the analysis of specific regions of interests from electronic health records images; mining diagrams can be useful for analysis of images with biological networks and embedded structural properties. Other than the mentioned ones, there are many domains which require bioimaging informatics e.g. *PCR-ELISA, microarray, DNA/RNA sequencing, diagnostic imaging CT/MRI, ultrasound scans, and medicinal imaging like EEG, MEG, ECG, PET and other anatomical or pathological images*. Moreover, an approach is needed that can analyse images based on clinical and genomic data, and help identify the information about disease causing genes with reference to the published literature. During this study, we have also tried to see which of the implemented approaches are well used by the communities. While conducting this study we faced some problems in finding explicitly provided information on the major limitations and developmental key elements, e.g. most of the authors published their work with open access publishers but did not provide the information about used tools and technologies and even did not provide the web links to the running systems or open access source code. Unfortunately, most of the published work is not implemented as public web/online system, so a normal user can use and evaluate at regular basis. We believe that it is important to have a real time user feedback in implementing such systems because if there will be not many people who can use it, then somewhat it will impairs the impact of research and development based on months and years of efforts. Moreover, we suggest that the bioimaging and NLP community should publish and provide their implemented methodologies in such forms; so then others do not need to spend much time in evaluating or rewriting the source code. Writing open access libraries, e.g. DLLs etc., including open source with good documentation and design, can also be helpful.

**Table 2.** Comparative analysis of bioimaging informatics approaches

Features/Approaches	Methodology Categorization (Image mining, text mining, Image and text mining)	Domain Categorization (Open, specific)	Web Links
Fiji (36)	Image Mining	Specific for electron microscopy data	http://fiji.sc/Fiji
Particle swarm optimization (39)	Image and text mining	Open for all kinds of images.	Not publically available online.
Figure panel classification (42)	Image mining	Open for all kinds of images.	Not publically available online.
Analyzing axis diagrams (50)	Image and text mining	Open for all kinds of axis diagrams.	Not publically available online.
Automatic categorization of biomedical images (45)	Image mining	Open for all kinds of flow charts, experimental, graph and mixed images	Not publically available online.
Yale Image Finder (48)	Image mining	Open for all kinds of biomedical images	http://krauthammerlab.med.yale.edu/imagefinder/
Hybrid framework (38)]	Image and text mining	Specific for protein-protein interaction images.	Not publically available online.
Low-level feature extraction with ontology (40, 41)	Image and text mining	Open for all kinds of images clinical health care images.	Not publically available online.
Mining images for the detection and analysis of gel diagrams (43)	Image and text mining	Specific for protein-protein interaction images.	Not publically available online.
Mining pathway diagrams (44)	Image and text mining	Specific for pathways analysis images.	www.biologicalnetworks.org
Edge-based image feature descriptor (47)	Image mining	Open for all kinds of images health care images.	Not publically available online.
Integrating image data into biomedical text categorization (49)	Image and text mining	Open for all kinds of biomedical images	Not publically available online.

During this study, we also found that one of the very commonly reported limitations for the researchers is the access to the published biomedical literature. Most of the biomedical literature is only available in restricted form and images are not well structured, which ultimately reduces access to the published literature. Moreover, almost all publishers are following different criteria for online and print publications, which also needs to be completely standardized. We observed that along with other technical and biological changes, one of the key challenges in biomedical image mining is the development of robust algorithms to analyse complex and heterogeneous images (121).

There are a number of databases, which directly profit from the discussed approaches in having higher quality data by applying such image mining tools. For instance, in the STRING database of protein interactions (and in many related databases), text mining can be applied for interaction predictions. However, here, reader often do not know whether this is just a discussion of the interaction, e.g. in the discussion section of the paper or whether there is a results figure, which gives firm proof of the interaction occurring according to experimental data. For such tasks, our tool (122) can ideally be suited, as it readily distinguishes and mines separately text from the main article and distinguishes it from figure legends and concrete results. Data mining tools and databases, which strongly profit from such data extraction tools to separate and distinguish among images, legends and text, include, for instance the i-HOP, where a separation between information ‘extracted from a text part’ and ‘extracted from a image part/so from original data’ is powerful and meaningful. Moreover, in scientific literature repositories and data warehouses, integration of efficient approaches for the extraction of text, images and text descriptions from images is important for the implementation of valuable information retrieval systems as well as for further improvement of the database and its content, e.g. *DrumPID* (123).

Other such situations include databases for biomedical images e.g. databases on microscopic images by The European Advanced Light Microscopy Network (EAMNET) or in particular the Image Browser by EMBL etc., where not only images are stored but very often secondary data including information about experimental settings and conditions is managed. To curate and improve such databases, tools to separate image-based information, legends and normal text in result sections are important as otherwise the information pertaining to the figures (including their legends) is not properly separated from text parts (explaining experimental conditions for the images or giving biological conclusions).

Finally, we also have for instance several imaging facilities at University of Würzburg (e.g. http://www.super-resolution.biozentrum.uni-wuerzburg.de/research_topics/, http://www.rudolf-virchow-zentrum.de/en/research/central-technologies/imaging.html). However, for the further development, a virtual research environment, i.e. a database storing and linking molecular data with images would be highly desirable. This can only be achieved and established if first the mixture of text, protocols and omics data is properly separated from images, figures and figure legends—again a task for which our tool (122) is perfectly suited. As, for the different use-cases and databases for which such approaches can be applied to illustrate that there are a number of situations where such tools are very useful. Combinations of such tools are obviously more powerful then stand-alone routines. However, of course there are possible alternative solutions, in particular regarding the implementation and separation of text and images in biological databases, which can be similar performant by design, e.g. by design of the database and regarding reproducible retrieval of the same text or image item from the same or a similar text, image with figure legend document. The reproducibility (124) and reliability of the overall data or database constructed from these tools depends on many other such database mining-tools, with the amount of additional curation done, in particular time-consuming curation by hand. For different use-cases and databases for which such approaches can be applied, we illustrate that there are a number of situations where such tools are very useful.

## Conclusion

In summary, our focussed review on such image and text mining tools is not a database in itself but quite instrumental and useful to develop all these above-mentioned databases and database types further and we are already using such approaches for this purpose in our own in-house databases.

## Supplementary data

Supplementary data are available at *Database* Online.

Supplementary Data
