# Supplementary material for: Mining biomedical images towards valuable information retrieval in biomedical and life sciences
Source: Database (Oxford). 2016 Aug 18;2016:baw118. doi: 10.1093/database/baw118 (PMC4990152; doi:10.1093/database/baw118)
Supplement: Supplementary Data [file supp_2016_baw118_index.html]

Mining biomedical images towards valuable information retrieval in biomedical and life sciences — Supplementary Data 

# Mining biomedical images towards valuable information retrieval in biomedical and life sciences

## Supplementary Data

files

- Supplementary Data - zip file
