# Supplementary material for: Mining biomedical images towards valuable information retrieval in biomedical and life sciences
Source: Database (Oxford). 2016 Aug 18;2016:baw118. doi: 10.1093/database/baw118 (PMC4990152; doi:10.1093/database/baw118)
Supplement: Supplementary Data [file supp_baw118_suppl_data.zip › SM_Table_S1.docx]

### Table. S1: *Databases for biomedical keeping the images of various disciplines.*

| ***Databases*** | **Web links** |
| --- | --- |
| *The Electron Microscopy Data Bank (EMDB) at PDBe* | (<https://www.ebi.ac.uk/pdbe/emdb/>) |
| *Whitney Imaging Center* | (<https://www.mycdi.com/whitney_imaging_center/>) |
| *COllaborative Informatics and Neuroimaging Suite (COINS)* | (<https://portal.mrn.org/micis/index.php?subsite=dx>) |
| *LONI image data archive* | (<https://ida.loni.usc.edu/services/Menu/IdaData.jsp?project>=) |
| *The Cancer Imaging Archive (TCIA)* | (<http://www.cancerimagingarchive.net/>) |
| *Grand Challenges in Medical Image Analysis* | (<http://www.grand-challenge.org/index.php/Main_Page>) |
| *Alzheimer’s Disease Neuroimaging Initiative (ADNI)* | (<http://adni.loni.ucla.edu/>) |
| *The Open Access Series of Imaging Studies (OASIS)* | (<http://www.oasis-brains.org/>) |
| *Breast Cancer Digital Repository (BCDR)* | (<http://bcdr.inegi.up.pt/>) |
| *Digital Database for Screening Mammography (DDSM)* | (<http://marathon.csee.usf.edu/Mammography/Database.html>), |
| *The Mammographic Image Analysis Society (MIAS)* | (<http://peipa.essex.ac.uk/info/mias.html>) |
| *Mammography Image Databases (MID)* | (<http://marathon.csee.usf.edu/Mammography/Database.html>) |
| *NLM HyperDoc Visible Human Project color, CAT and MRI image samples* | (<http://www.nlm.nih.gov/research/visible/visible_human.html>) |
| *The Histology Image Dataset (histologyDS)* | (<http://www.informed.unal.edu.co/histologyDS>) |
| *The Cancer Genome Atlas (TCGA)* | (<http://cancergenome.nih.gov/>) |
| *International Cancer Genome Consortium (ICGC)* | (<http://icgc.org>) |
| *Stanford Tissue Microarray Database (TMA)* | (<http://tma.im>) |
| *MITOS dataset* | (<http://ipal.cnrs.fr/ICPR2012/>) |
| *Cancer Image Database* (caIMAGE) | (<http://emice.nci.nih.gov/caimage>) |
| *DPA’s Whole Slide Imaging Repository* | (<https://digitalpathologyassociation.org/whole-slide-imaging-repository>) |
| *Atlas of bleast Histology* | (<http://www.webmicroscope.net/atlases/breast/brcatlas_start.asp>) |
| *Histology Photo Album* | (<http://www.histology-world.com/photoalbum/thumbnails.php?album=52>) |
| *Tissue Acquisition and Banking Services (TABS) of the NYU Experimental Pathology Core Facilities* | (<http://pathology.med.nyu.edu/research/core-laboratories/tissue-banking>) |
| *Aperio Images* | (<http://images2.aperio.com/>) |
| *HAPS Histology Image Database* | (<http://hapshistology.wetpaint.com/>) |
| *ITK Analysis of Large Histology Datasets* | (<http://www.na-mic.org/Wiki/index.php/ITK_Analysis_of_Large_Histology_Datasets>) |
| *BDGP images from the FlyExpress database* | ([www.flyexpress.net](http://www.flyexpress.net)) |
| *The UCSB Bio-Segmentation Benchmark dataset* | (<http://www.bioimage.ucsb.edu/research/biosegmentation>) |
| *Pap Smear database* | (<http://labs.fme.aegean.gr/decision/downloads>) |
| *BIICBU Biological Image Repository* | (<http://ome.grc.nia.nih.gov/iicbu2008/>) |
| *RNAi dataset* | (<http://ome.grc.nia.nih.gov/iicbu2008/rnai/index.html>) |
| *Chinese Hamster Ovary cells (CHO) dataset* | (<http://ome.grc.nia.nih.gov/iicbu2008/hela/index.html#cho>) |
| *Locate Endogenus mouse sub-cellular organelles (END)* database | (<http://locate.imb.uq.edu.au/>) |
| *2D HeLa dataset (HeLa) database* | (<http://ome.grc.nia.nih.gov/iicbu2008/hela/index.html>) |
| *Allen Brain Atlas* | (<http://www.brain-map.org/>) |
| *1000 Functional Connectomes Project* | (<http://fcon_1000.projects.nitrc.org/>) |
| *The Cell Centered Database (CCDB)* | (<http://ccdb.ucsd.edu/index.shtm>) |
| *The Encyclopedia of DNA Elements (ENCODE)* | (<http://genome.ucsc.edu/ENCODE/>) |
| *The Human Protein Atlas* | (<http://www.proteinatlas.org/>) |
| *DRIVE: Digital Retinal Images for Vessel Extraction* | (<http://www.isi.uu.nl/Research/Databases/DRIVE/>) |
| *El Salvador Atlas of Gastrointestinal Video Endoscopy Images and Videos of his-res of studies taken from Gastrointestinal Video endoscopy* | (<http://www.gastrointestinalatlas.com/>) |
| *BiMed* | (<https://sites.google.com/site/aacruzr/projects/bimed>) |
| *Public Image Databases* | (<http://www.via.cornell.edu/databases/>) |
| *Dartmouth Biomedical Libraries* | (<http://www.dartmouth.edu/~library/biomed/guides/research/medimages.html?mswitch-redir=classic>) |
| *The National Library of Medicine presents MedPix* | (<https://medpix.nlm.nih.gov/home>) |
| *New Database Provides Millions of Biomedical Images* | (<http://info.hsls.pitt.edu/updatereport/?p=4402>) |
| *DrumPID* | (<http://drumpid.bioapps.biozentrum.uni-wuerzburg.de/compounds/index.php>) |
| *STRING* | (<http://string-db.org/>) |
