# Supplementary material for: Mining biomedical images towards valuable information retrieval in biomedical and life sciences
Source: Database (Oxford). 2016 Aug 18;2016:baw118. doi: 10.1093/database/baw118 (PMC4990152; doi:10.1093/database/baw118)
Supplement: Supplementary Data [file supp_baw118_suppl_data.zip › SM_Table_S2.docx]

**Table. S2:** *MEDLINE citation count taken from the official website (*[*http://www.nlm.nih.gov/bsd/medline_cit_counts_yr_pub.html*](http://www.nlm.nih.gov/bsd/medline_cit_counts_yr_pub.html)*) of the MEDLINE by the US national Library of Medicine (as of mid - November 2015)**

| **Years of Publication** | **Total Number of Citations** |
| --- | --- |
| Pre-1947 | 41,755 |
| 1947 | 38,293 |
| 1948 | 41,292 |
| 1949 | 49,566 |
| 1950 | 82,084 |
| 1951 | 95,885 |
| 1952 | 94,269 |
| 1953 | 92,155 |
| 1954 | 87,777 |
| 1955 | 89,110 |
| 1956 | 86,153 |
| 1957 | 88,719 |
| 1958 | 86,258 |
| 1959 | 93,545 |
| 1960 | 100,336 |
| 1961 | 107,166 |
| 1962 | 115,572 |
| 1963 | 136,537 |
| 1964 | 156,748 |
| 1965 | 171,771 |
| 1966 | 175,070 |
| 1967 | 186,903 |
| 1968 | 203,763 |
| 1969 | 210,583 |
| 1970 | 212,447 |
| 1971 | 217,566 |
| 1972 | 222,189 |
| 1973 | 225,879 |
| 1974 | 229,846 |
| 1975 | 244,085 |
| 1976 | 249,193 |
| 1977 | 255,748 |
| 1978 | 265,557 |
| 1979 | 274,295 |
| 1980 | 272,513 |
| 1981 | 274,458 |
| 1982 | 285,155 |
| 1983 | 299,031 |
| 1984 | 307,940 |
| 1985 | 318,101 |
| 1986 | 330,463 |
| 1987 | 348,009 |
| 1988 | 365,617 |
| 1989 | 381,408 |
| 1990 | 388,194 |
| 1991 | 388,803 |
| 1992 | 391,828 |
| 1993 | 398,106 |
| 1994 | 407,330 |
| 1995 | 416,500 |
| 1996 | 421,876 |
| 1997 | 432,049 |
| 1998 | 446,885 |
| 1999 | 459,667 |
| 2000 | 485,456 |
| 2001 | 505,641 |
| 2002 | 521,232 |
| 2003 | 548,766 |
| 2004 | 578,675 |
| 2005 | 608,943 |
| 2006 | 633,960 |
| 2007 | 656,794 |
| 2008 | 685,103 |
| 2009 | 706,217 |
| 2010 | 731,760 |
| 2011 | 762,959 |
| 2012 | 802,026 |
| 2013 | 809,636 |
| 2014 | 811,002 |
| 2015 | 246,372 |
